# Ultra-low radiation dose protocol for CT-guided intrathecal nusinersen injections for patients with spinal muscular atrophy and severe scoliosis

**DOI:** 10.1007/s00234-021-02643-9

**Published:** 2021-01-29

**Authors:** Grzegorz Rosiak, Anna Lusakowska, Krzysztof Milczarek, Dariusz Konecki, Anna Fraczek, Olgierd Rowinski, Anna Kostera-Pruszczyk

**Affiliations:** 1 II Department of Radiology, Warsaw Medical University, Banacha 1a, 02-097 Warszawa, Poland; 2 Department of Neurology, Warsaw Medical University, Banacha 1a, 02-097 Warszawa, Poland

**Keywords:** Spinal muscular atrophy, SMA, CT-guided injections, Nusinersen

## Abstract

**Purpose:**

Intrathecal injection of nusinersen is an approved treatment of spinal muscular atrophy (SMA). CT-guided injection is a method of nusinersen administration in patients with severe scoliosis, in whom standard lumbar puncture is not feasible. The injections are repeated every 4 months for life, and accumulated radiation doses absorbed by the patient can increase the risk of cancer. In this study, we present the results of CT-guided intrathecal nusinersen injections with an ultra-low radiation dose protocol.

**Methods:**

Eighteen patients (15 adults and three children) in whom standard lumbar puncture was not feasible due to severe scoliosis or spinal stabilization were included in this retrospective study. The first 23 injections were performed with a standard radiation dose protocol and the next 42 injections with an ultra-low-dose protocol. The radiation doses, measured as total dose length product (DLP), were acquired and compared between the protocols.

**Results:**

Injections were successful in 100% of patients with both ultra-low-dose and standard protocols. The radiation dose, measured as DLP, was 111.2–1100.7 (*Me* = 248.1) mGy*cm for the standard protocol. For the ultra-low-dose protocol, the dose range was 5.0–54.4 (*Me* = 26.7) mGy*cm, which was significantly lower than with the standard protocol (*p* < 0.001, *η*^2^ = 0.67).

**Conclusion:**

Radiation doses can be significantly decreased in the CT-guided injection of nusinersen. The proposed protocol allows for effective CT-guided intrathecal nusinersen administration in patients with SMA and severe scoliosis.

## Introduction

Spinal muscular atrophy (SMA) is a hereditary disease characterized by progressive muscle weakness and atrophy due to the degeneration of motor neurons caused by mutations in the *SMN1* gene [1]. Nusinersen is the first therapeutic agent to be approved for the treatment of SMA. It does not cross the blood-brain barrier and has to be administered intrathecally. However, in some patients, intrathecal drug injection via standard lumbar puncture (without imaging guidance) is not possible due to anatomical distortion of the spine (e.g., severe scoliosis with rotation or presence of stabilization instruments). Computed tomography (CT)–guided intrathecal injection is an effective method for drug delivery in these patients [2, 3].

After treatment induction (four doses within 2 months), patients with SMA require three nusinersen injections annually; for those with severe scoliosis, this means multiple CT-guided procedures throughout their lifetime. A high number of CT scans increase the risk of cancer in a cumulative manner due to the high radiation doses absorbed by patients [4]. There is no dose threshold above which the carcinogenic effects are present; however, the carcinogenic effects are dose-dependent, meaning that the risk accumulates with every radiation-based imaging study. This is an even greater issue in children, who are more sensitive to radiation in terms of the development of cancer [5]. Therefore, the reduction of radiation doses is an important issue, especially in patients requiring multiple CT scans annually.

CT has excellent spatial resolution, which allows spinal interventional procedures to be performed with great accuracy [6]. It is particularly useful in intrathecal injections requiring high-quality imaging of bones and soft tissues.

CT-guided procedures usually involve helical scans and intermittent or “quick-check” scans. The quick-check CT fluoroscopy method allows three contiguous images to be displayed in a single pedal step. This significantly reduces radiation during the procedure due to the short exposition time [7]. Patients absorb more than 90% of the radiation dose during helical scans [8], so reduction of these scans is crucial to minimize radiation doses.

The few studies reporting radiation doses during CT-guided nusinersen injections [3, 9–11] reported doses ranging from 85 to 173 mGy*cm. In our study, we tested an ultra-low-dose CT scan protocol with reduced tube current values and scanning area to decrease the radiation doses. The secondary purpose of the study was to assess the technical success rate of nusinersen administration by CT-guided injection in patients with scoliosis and/or instrumentation.

## Materials and methods

**Fig. 1:**
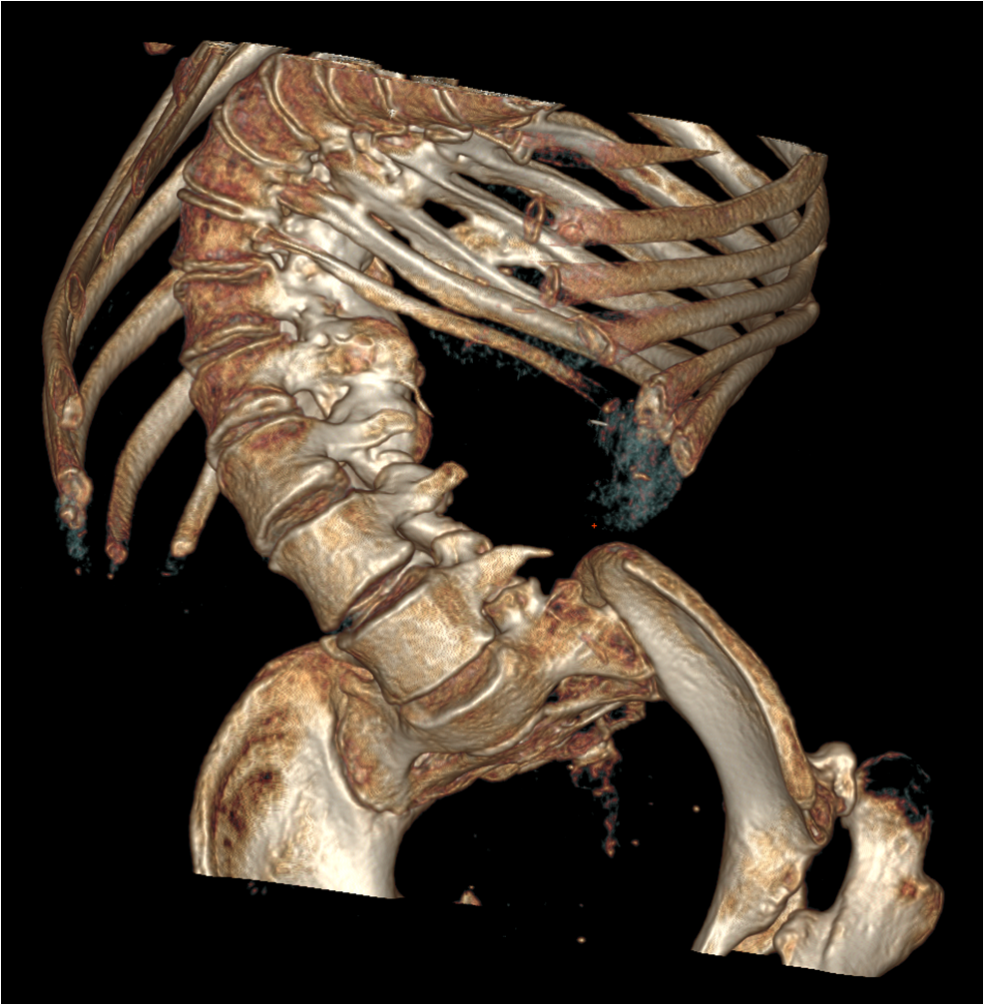
Severe scoliosis in a patient with SMA—volume rendering reconstruction

Eighteen patients (15 adults and three children; eight with type 1 SMA, seven with type 2 SMA, and three with type 3 SMA) who received CT-guided nusinersen injections in 2019 at our department were included in the study. The age of patients ranged from 8 to 47 years (mean 27.5). Standard lumbar puncture was not feasible due to the presence of severe scoliosis (Fig. 1) or stabilization devices in all patients. The Warsaw Medical University Bioethical Committee approved the study.

**Fig. 2:**
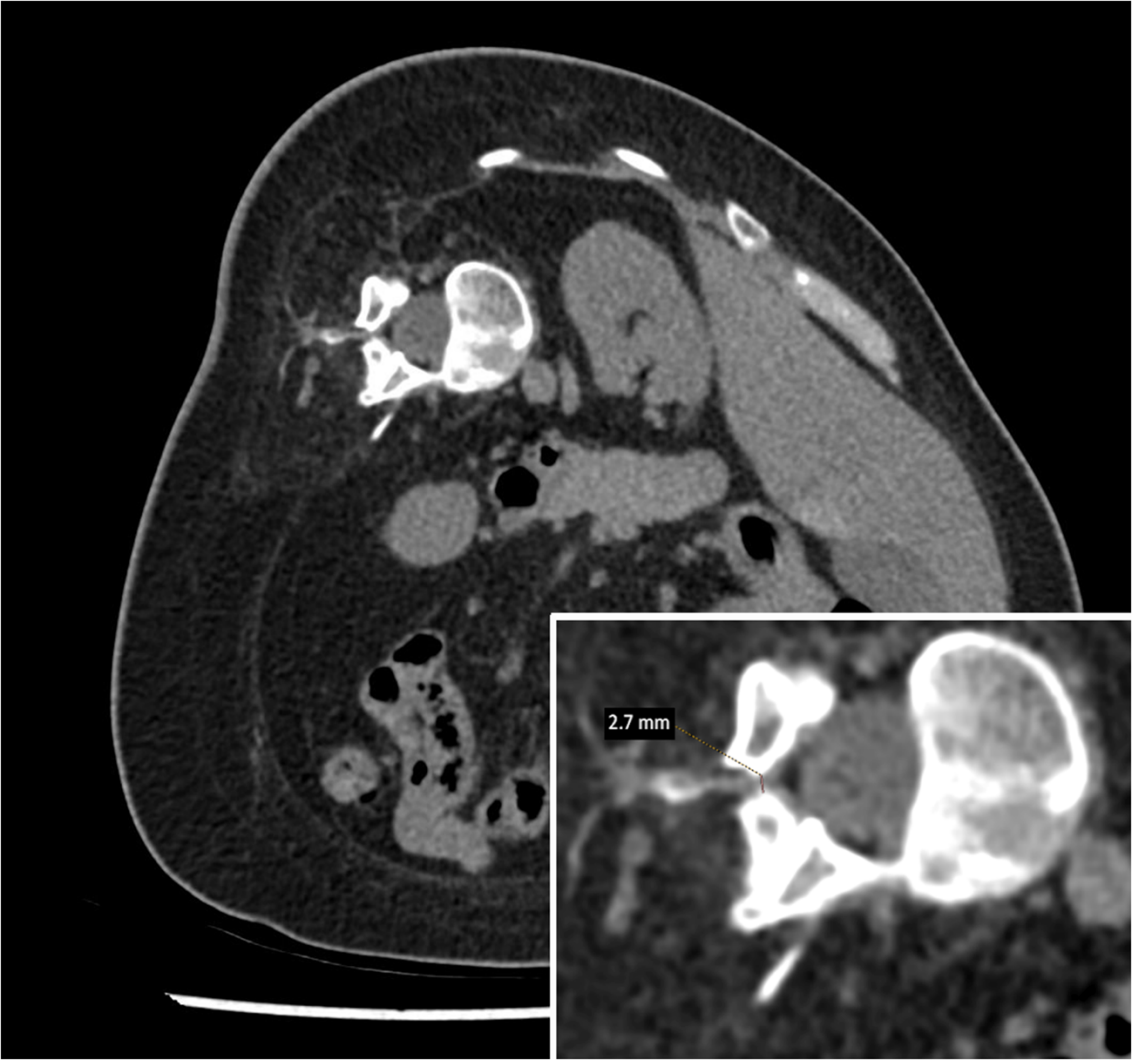
Narrow (2.7 mm) entry for interlaminar access

Sixty-five CT-guided injections of nusinersen were performed during the study period. The injections were performed at the interventional CT suite, using CT scanner Aquilion One (Toshiba/Canon, Nasu, Japan), by interventional radiologists with at least 5 years of experience in CT-guided procedures. Interlaminar access was used when the access window was at least 2 mm in diameter (Fig. 2); otherwise, the injection was performed via transforaminal access.

In three patients, qualification for the CT-guided procedure was done by performing an L-S spine CT exam 3–4 weeks before the injection. All patients were accepted for the CT-guided procedures, which were successful in all cases.

EMLA cream was applied on the skin before 47 injections. The three children included in the study received a mild sedative (hydroxyzine 3–8 ml, 2 mg/ml) before the procedure. Two adult patients required mild sedation with diazepam (5 mg). The patients were placed in the left lateral position. Antiseptic liquid was applied on the skin at the predicted needle entry site, followed by sterile draping.

In the first 23 injections, the standard method applied in other CT-guided procedures (e.g., biopsies, drainages, or ablations) was used. First, a helical scan of the lumbar spine was done (100 mA, 120 kV). The length of the scanned area was about 20 cm. The needle placement was performed under CT guidance with a quick-check method, followed by a post-injection helical scan for potential adverse events.

The next 42 injections were performed according to the ultra-low-dose protocol. After the acquisition of two scout images, a helical scan targeted at the expected area of injection was done. The length of the scanned area was 4–12 cm, depending on the expected difficulty in choosing an appropriate injection level. A current of 10 mA was applied, as opposed to the 100 mA current used in a standard CT scan of the lumbar spine.

**Fig. 3:**
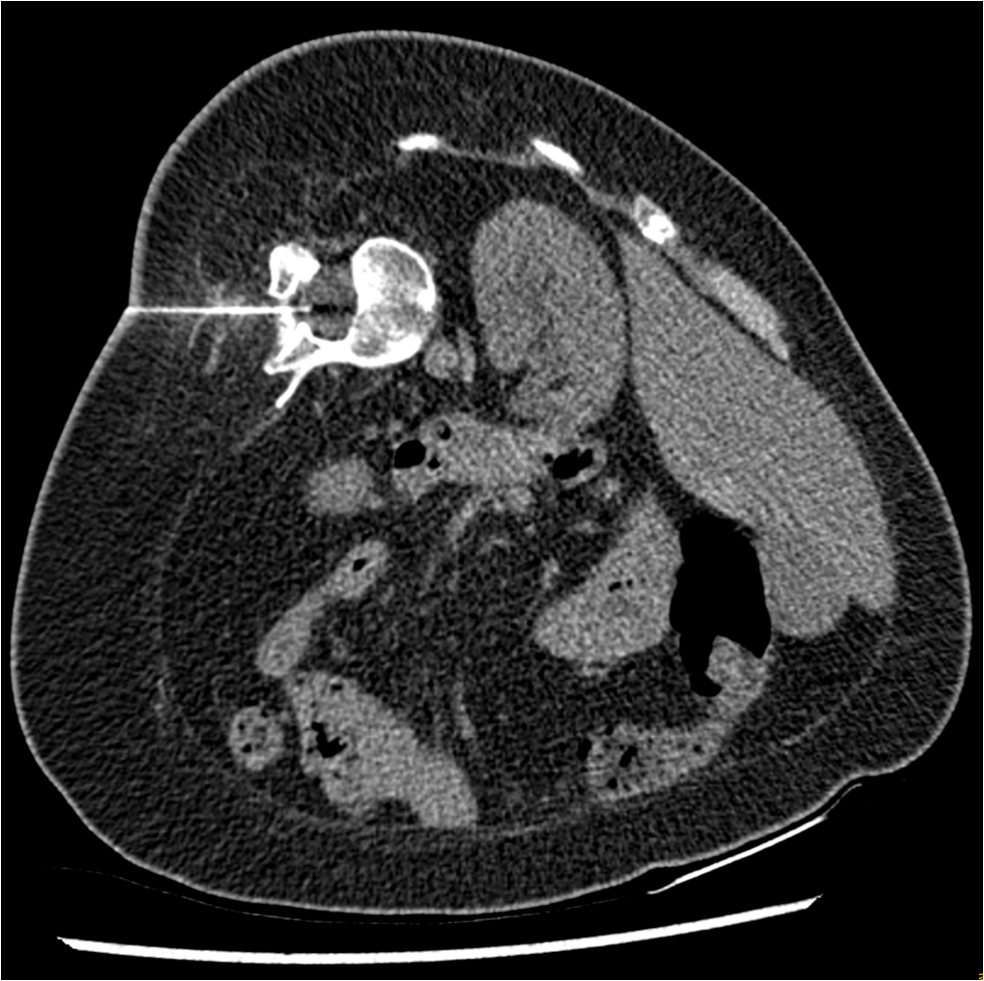
CT-guided intrathecal nusinersen injection—standard protocol

**Fig. 4:**
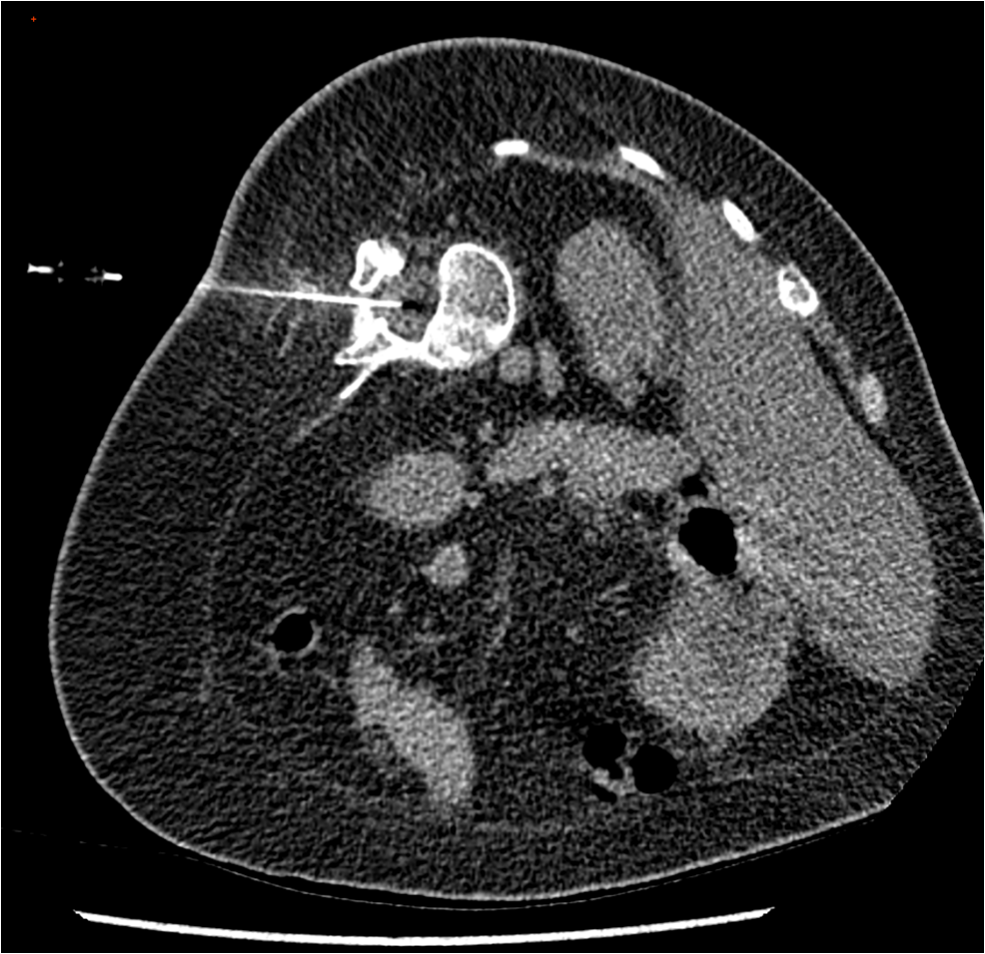
CT-guided intrathecal nusinersen injection—ultra-low-dose protocol

In both protocols, the needle trajectory was planned according to the initial scan, and the lumbar puncture needle entry site was marked with a short, thin needle. The short needle position was verified with a quick-check scan and corrected if needed. Then, the 23G spinal needle (BD, Oxford, UK) was inserted, followed by a quick-check scan after each progression of the needle. After positioning the tip of the needle intrathecally, the stylet was withdrawn, and 5 ml of cerebrospinal fluid was removed. Then, 12 mg of nusinersen (5 ml) was injected over 1–2 min. The quality of the scans is presented in Fig. 3 (standard protocol) and Fig. 4 (ultra-low-dose protocol).

In the new, ultra-low-dose protocol, the distance between the needle entry site and the intergluteal cleft or a nevus was measured during the first nusinersen injection. In the following injections, the scanned area was limited to the region selected during the first procedure, thus decreasing the radiation dose. After removal of the needle, a final quick-check scan was performed to visualize the area of injection for possible adverse events (e.g., bleeding or extrathecal injection).

CT-guided nusinersen administration was classified as successful if the tip of the needle was placed intrathecally (confirmed on the CT scan), there was cerebrospinal fluid outflow from the needle, the whole 5 ml of nusinersen was injected with even resistance, and there was no extrathecal fluid visible around the thecal sac after injection.

The procedures were divided into 2 groups according to the CT scan protocols applied during the procedures: standard protocol and low-dose protocol (details of the protocols are in Table 1). The radiation doses (measured as total dose length product—DLP) were acquired from the imaging dose reports delivered by the scanner after each procedure. The significance of difference between the quantitative variables (age and procedure time) was analyzed with Mann-Whitney *U* test and calculated chi^2^ (or Fisher exact test in case of small expected values) for qualitative variables (spine level selected and sedation) (Tables 2 and 3). Then, the Mann-Whitney *U* test was used to calculate the significance of difference between DLP. The statistics of dose in each group were placed in Table 4.

**Table 1:** Standard and ultra-low-dose protocols

	Standard protocol	Ultra-low-dose protocol
Current	100 mA	10 mA
Voltage	120 kV	120 kV
Slice thickness	0.5–0.8 mm	0.5–0.8 mm
Rotation time	500 ms	500 ms
Scan length	18–29 cm	4–12 cm

**Table 2:** Mann-Whitney *U* test results for comparisons of age and procedure time in groups with low- and standard dose protocols

	Low-dose protocol (*n* = 42)			Standard dose protocol (*n* = 23)			*p*
	Me	25th percentile	75th percentile	*Me*	25th percentile	75th percentile	
Age	28.50	22.00	31.00	32.00	24.00	34.00	0.051
Procedure time	23.00	19.00	30.25	28.00	24.00	31.00	0.019

*Me* median, *p p* value for Mann-Whitney *U* test

**Table 3:** Frequencies of spine level selected (Fisher exact test) and sedation (chi^2^ test) results in groups with low- and standard dose protocols

	Low-dose protocol (*n* = 42)		Standard dose protocol (*n* = 23)		*p* value
	Number	Percent	Number	Percent	
Spine level selected					0.801
L2/L3	4	9.5	3	13.0	
L3/L4	11	26.2	4	17.4	
L4/L5	12	28.6	6	26.1	
L5/S1	15	35.7	10	43.5	
Sedation					0.243
Yes	15	35.7	5	21.7	
No	27	64.3	18	78.3

**Table 4:** Statistics of doses (mGy*cm) between standard and ultra-low-dose protocols

	Mean	Median	25th percentile	75th percentile	SD	Min.	Max.
Ultra-low-dose protocol (*n* = 42)	27.46	26.70	18.38	34.88	12.46	5.00	54.40
Standard protocol (*n* = 23)	365.77	248.10	190.50	462.90	253.94	111.20	1100.70

*SD* standard deviation, *Min.* minimum, *Max*. maximum

## Results

Of the 18 patients included in the study, 16 patients presented with severe scoliosis, and two had spinal stabilization making standard lumbar puncture impossible to carry out. Of the 65 injections performed, 60 were performed via standard posterior access in 16 patients, and five injections in two patients were performed via transforaminal access. None of the patients required laminectomy. In 100% of procedures, the needle was placed intrathecally, and injections were successful. Intrathecal administration was confirmed with a post-procedural CT scan in which no extrathecal fluid was visible.

The groups differed in the duration of the procedure—in the group with lower doses, the time of the procedure was significantly shorter, but the effect size was moderate (*Me* = 23.00 vs *Me* = 28.00)—M-W *U* test; *p* = 0.019, *η*^2^ = 0.08. Age differences turned out to be statistically insignificant (M-W *U* test; *p* = 0.051, *η*^2^ = 0.06) as well as frequencies of the selected spinal level (Fisher exact test: *p* = 0.801) and sedation (chi^2^: *p* = 0.243).

**Fig. 5:**
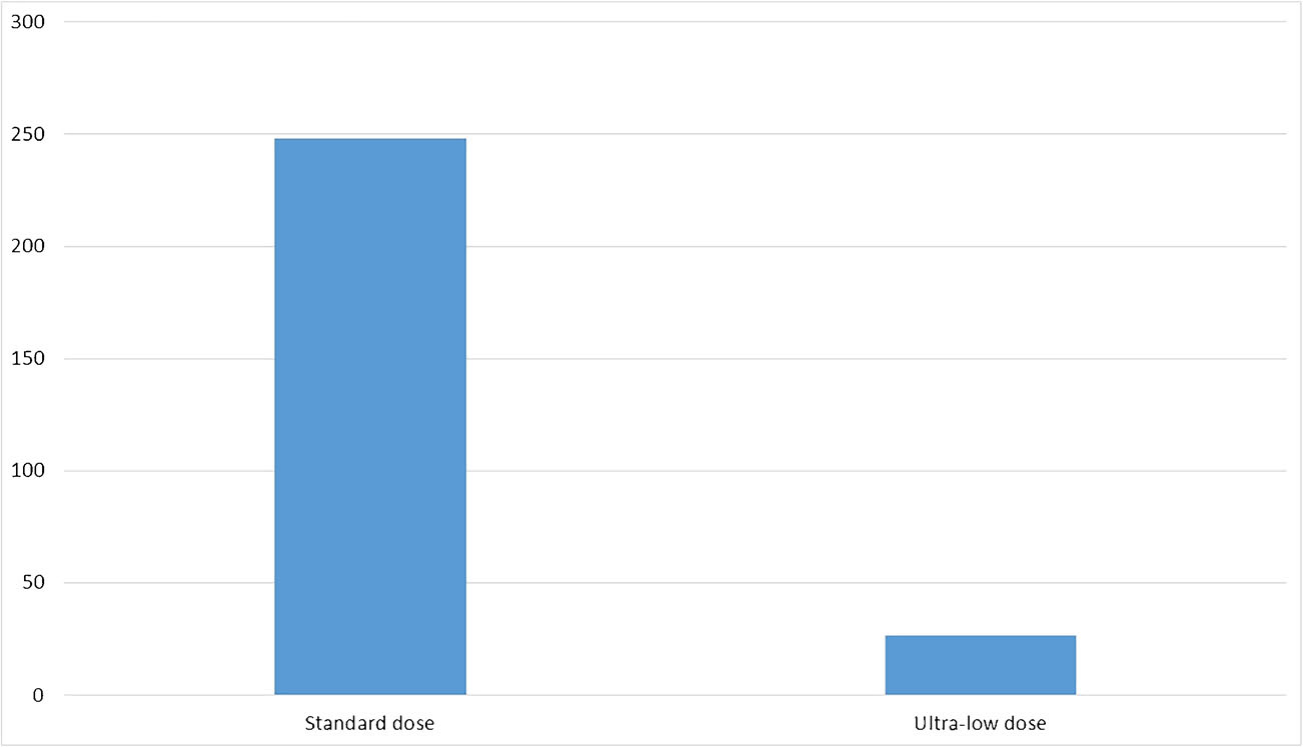
Median dose in group with low-dose protocol and standard protocol

The radiation dose measured as DLP was 111.2–1100.7 (*Me* = 248.1) mGy*cm for the standard protocol (Table 4). For the ultra-low-dose protocol, the dose range was 5.0–54.4 (*Me* = 26.7) mGy*cm, which was significantly lower than for the standard protocol (*p* < 0.001, *η*^2^ = 0.67) (Fig. 5). The DLP values at the 75th percentile were 462.9 mGy*cm for the standard protocol and 34.88 mGy*cm for the ultra-low-dose protocol.

One patient experienced a headache after the first injection performed with standard protocol that resolved within 24 h (1.5% of 65 scans). No other minor or major complications were noted during or after the procedure.

## Discussion

CT-guided intrathecal injection is a feasible method of nusinersen administration for patients in whom standard lumbar puncture is not possible due to severe scoliosis or spinal stabilization. In most patients, CT-guided procedures such as biopsies or ablations are performed once in their lifetime, and radiation doses are relatively small [8]. However, patients with SMA will have CT-guided procedures done at least three times per year, resulting in a high total number of CT scans and high accumulated radiation dose. The carcinogenic effect of radiation is well documented [4] and is even more prominent in pediatric patients [12]. Therefore, radiation doses from imaging studies should be kept as low as reasonably achievable (ALARA rule), especially in patients who undergo repeated scans.

The achievable dose (AD) represents the 50th percentile (median) of the dose distribution for the imaging study, meaning that the radiation dose should be lower than the AD in 50% of the procedures. The AD for CT-guided nusinersen injections proposed by Oldenburg et al. [10] was 120.1 mGy*cm in terms of DLP. The diagnostic reference level (DRL) represents the 75th percentile of the dose distribution and was set at 233.1 mGy*cm by Oldenburg et al. [10]. The results of our study show that radiation doses can be much lower. When the ultra-low-dose protocol was used, the 75th percentile (corresponding to the DRL) was 34.3 mGy*cm, and the median DLP value (corresponding to the AD) was 26.7 mGy*cm.

The doses (in terms of DLP) for spinal CT-guided interventions published by Guberina et al. [13] ranged from 460 to 620 mGy*cm. Studies of radiation doses in CT-guided intrathecal nusinersen injections have reported a DLP of 52.1 [11] to 85.6 mGy*cm [3]. In the study by Stolte et al. [14], the average dose was 2.6 mSv, which converts by a factor of 0.015 [15] to approximately 173 mGy*cm. In the study by Bortolani et al. [2], the mean radiation dose was 2.15 mSv per patient, which is equivalent to 143.3 mGy*cm.

Interlaminar approach with fluoroscopic guidance is a safe and effective method for reaching the intrathecal space; it provides real-time images and better control of the needle progression. It allows for successful nusinersen injection in difficult patients [16] and is widely used especially in centers where CT suites are not freely available for interventions. Jacobson et al. [17] used conventional fluoroscopy guidance in transforaminal nusinersen injections and reported the mean effective dose as 1.07 mSv, which corresponds to 71 mGy*cm (2.5 times higher than in our ultra-low-dose protocol). These numbers do not show an advantage of conventional fluoroscopy over CT guidance in terms of radiation exposure. Additionally, the report by Bortolani et al. [2] showed a higher complication rate (15%) with transforaminal access than with the interlaminar (5%) approach. In our study, one patient presented with a headache after one procedure (1.5%) that resolved within 24 h, and no other complications were reported.

In the study by Kizina et al. [9], the mean dose area product (DAP) was 200.48 μGym^2^ which after application of conversion coefficients would correspond to around 31 mGy*cm (slightly more than in our ultra-low-dose protocol).

The reports on cone-beam CT guidance in terms of nusinersen injection [18, 19] present high technical success rate. However, the authors report transforaminal access only which makes it difficult to compare with interlaminar approach. Also, in one case, large bowel was punctured which would probably not happen with high-quality CT guidance.

In our study, all patients had a helical CT scan done just before the first injection, which was sufficient to plan a needle trajectory. There was therefore no added value in an earlier pre-procedural scan (days or weeks before the injection) as it can be done immediately before the procedure. No extrathecal injection was detected so post-injection imaging can probably be eliminated to further reduce the dose. However, this should be confirmed in a larger group.

The procedure duration time in ultra-low-dose protocol was shorter than in standard protocol. This fact could be owed to higher operators’ experience when performing ultra-low-dose protocol injections (standard dose procedures were done at the beginning of nusinersen injection program).

Radiation doses can be reduced by lowering the tube current and using shorter helical scans. Careful planning of consecutive CT-guided injections is crucial in terms of limiting the scan area and includes measuring the distance from the intergluteal cleft to the needle entry site. We also believe that a further reduction in radiation doses is possible in some patients by lowering the tube voltage from 120 to 100 kV.

Due to the 100% success rate of CT-guided injections in our study, none of the patients required laminectomy [20], and we believe that such an invasive procedure should be reserved for patients who cannot have a nusinersen injection under imaging guidance. Cervical puncture can be used for nusinersen administration [21]; however, this approach was not necessary due to the high technical success rate of lumbar punctures.

Apart from radiation safety, a chronic damage to thecal sac should be taken into consideration. Even though none of the patients in this study presented with CSF leak, such complication should not be unexpected in the long term.

The main limitation of the study is the relatively small number of patients, and further research on larger groups should be conducted. The learning curve may also have affected the number of scans done during the procedures.

## Conclusion

The results of our study show that, compared to previous reports, radiation doses can be significantly decreased in the CT-guided injection of nusinersen. The proposed protocol allows for a high technical success rate—100% of injections in the study were successful. An earlier CT scan does not seem necessary since all patients in whom standard lumbar puncture was not feasible were qualified for the CT-guided procedure.

## Abbreviations

mA: Milliampere (tube current)
kV: Kilovolt (tube voltage)
DLP: Dose length product
DRL: Dose reference level
AD: Achievable dose
SMA: Spinal muscular atrophy
